# A Novel Scheme of Re-Organisation

**Published:** 1920-05-29

**Authors:** 


### THE FUTURE HEALTH SERVICES.

### A Novel Scheme of Re-organisation.

The Consultative Council on Medical and Allied Services, which is associated with the Ministry of Health and was appointed in October last, was invited by Dr. Addison on its formation to consider the problem of forming a systematised medical service established on a local basis but applicable, area for area, to the whole country. The Council, of which Lord Dawson of Penn is the Chairman, has now issued an interim report, not, indeed, as a final exploration of so large and complicated a subject, but rather as an indication of the trend of its deliberations and conclusions up to date, As the complexity of treatment becomes greater, it grows increasingly difficult for the individual practitioner to administer the full range of treatment, while the number of patients who can afford to pay for it diminishes. Public opinion, again, appreciates more and more that the home does not always afford the best hygienic conditions for recovery from serious illness. The Council lays it down that any scheme of medical service must be open, though not necessarily free, to all classes of the community; that it must be such as can grow and expand and adapt itself to varying local conditions; and that in each locality it must comprise and provide for all the medical services, preventive and curative, necessary to the health of the people. All these agencies are to be brought together in close co-ordination under a single health authority for each area.

### The Centre of the Service.

At the centre of the medical service of the country lies the treatment which the medical practitioner gives to his patient, either at his own surgery or at the patient's private house. The report of the Consultative Council contemplates that this domiciliary medical service should continue. It would, however, be brought into relation with a Primary Health Centre, which would serve as the rallying-point of all the medical services, preventive and curative, of the district for which it was established. There would be at the Primary Health Centre, according to this outline, wards of varying sizes and for varying purposes, including provision for midwifery,, an operating-room, a radiography room, a laboratory for simple investigations, a dispensary, medical baths, and a common room, which would serve as a meeting-place for the general practitioners of the district and for the storage of clinical records on an agreed and standardised basis. A Primary Health Centre would also contain accommodation for communal and preventive services, such as those for pre-natal care, child welfare, medical inspection and treatment of schoolchildren, physical culture, and the examination of suspected cases of tuberculosis. So far as midwives and nurses are not available in particular districts under other arrangements, their services could be provided from a centre. A dental clinic with a staff of visiting dental surgeons would be another important branch of the equipment. It is suggested that in many instances existing buildings, such as cottage hospitals, could be adopted as Primary Health Centres, at any rate as a beginning.

### Primary and Secondary Centres.

It would be the distinguishing feature of these Primary Health Centres, one of which should ultimately be found in every convenient centre of population, that they should be staffed by the general practitioners of the district which they serve, patients who visited them or were accommodated in them retaining the services of their own doctors. The general practitioner would be able in a proper case to arrange for the transference of a patient to the Primary Health Centre where, retaining the patient still under his own care and control, he would be able to continue the treatment under more favourable circumstances and with a readier access to the resources of modern medical science than are afforded in the surgery or are possible within the patient's own home. The Primary Health Centre would provide the patient (on the terms described below) with food, nursing, and all equipment for efficient treatment, but not with medical attendance, which would be paid for either by the patient himself (if a private patient) or through some method of insurance or by the local health authority. While the Primary Health Centre thus provided the general practitioner with means not now generally available of offering his patient in a proper case what may be described as "hospital treatment" whilst still keeping him under his own control, it would also serve the general practitioner as a centre of professional life bringing him into daily contact with the other practitioners of his district and occasional contact with the consultants and specialists who would attend at fixed intervals from the Secondary Health Centres with which each group of Primary Centres would be brought into relationship.

The Secondary Health Centre of each district would be situated in a town where an adequate equipment would be possible and an efficient staff of consultants and specialists could be assembled. Each Secondary Health Centre would be within access of all the Primary Health Centres in the area. The Consultative Council contemplates that for many Secondary Centres the nucleus or organisation would be found in existing hospitals. In other districts, however, it would be necessary to establish a complete and model Secondary Health Centre. In this connection they point out the importance of a Hospital Survey at some early date. The results of this survey would afford statistical

**National Health Services**—(*continued*).

data for recognising the areas in which the existing provision is inadequate and the degree of the inadequacy. Secondary Health Centres would, in fact, need a complete hospital equipment.

Cases referred for consultation or treatment from the Primary Health Centres would attend at the out-patient clinics of the Secondary Centre or would occupy in-patient beds. The medical staff of the Secondary Centre would be responsible for the treatment of these cases, but general practitioners would have every opportunity to keep in touch with their patients while attending the Centre and to resume supervision over them on discharge. The duties of consultants attached to Secondary Centres would consist of regular attendance at fixed times in their out-patient clinics where they would see cases referred to them; periodical visits to Primary Health Centres in the district allotted to them and special visits of emergency to Primary Health Centres and in certain circumstances to the homes within their areas, always in consultation with the general practitioner. The consultants would be part-time officers and would be paid on a time basis with extra fees for special visits. This would leave them time for their private consulting practice. The report discusses at length the qualifications necessary for entrance into this consultant service and the methods of election to it by committees of selection on which medical men would have the larger representation. In those parts of the country where it is geographically possible it would be desirable that every Secondary Health Centre should be brought into relationship with the Teaching Hospital. The Teaching Hospital of the district would be found in some large city, and to it would go cases of unusual difficulty from Secondary and Primary Health Centres, which would in turn be permeated by the academic influence and the spirit of inquiry and progress associated with a Teaching Hospital.

It has already been pointed out that the Primary Centre would, according to the views of the Council, provide the patient with food, nursing, and all equipment for efficient treatment, but not with medical attendance. At the Secondary Centres the contribution of cost made by the patient would include in addition the services of the consultant, though it would be open for the patient to request the additional service of a selected consultant or specialist, being in this case responsible for the fee. On the subject of finance generally, the Consultative Council recognise that while preventive services must of necessity be publicly provided, the provision of curative services free of charge at the health centres would impose a heavy burden on public funds. While therefore certain members feel that these services should be provided at the cost of the community, the majority recommend that in the public wards of the Primary and Secondary Health Centres standard charges should be made for treatment, though it is contemplated that these charges might vary in different parts of the country, and that they could only as a rule be a contribution to the cost of treatment, which is often in its entirety beyond the means of many citizens. The Council further recommends that private and self-supporting wards should be a part of the provision at the health centres though the essential services in the public and private wards would be identical.

In order to administer the scheme in each district the Council glances at the need for a new type of local health authority to bring about unity of local control of all health services, curative and preventive. On this body the Council asks for due representation of the medical profession, and is of opinion that the authority should in each case be assisted by a Local Medical Advisory Council.

On the subject of a State Medical Service the Report says " the alternative of a whole-time salaried service for all doctors has received our careful consideration, and we are of opinion that by its adoption the public would be serious losers. No doubt laboratory workers and medical administrators who do not come in personal contact with the sick can with advantage be paid entirely by salary. The clinical worker, however, requires knowledge not only of the disease, but of the patient: his work is more individual, and if he is to win the confidence so vital to the treatment of illness, there must be a basis not only of sound knowledge, but of personal harmony. The voluntary character of the association between doctor and patient stimulates in the former the desire to excel both in skill and helpfulness. It is a true instinct which demands 'free choice of doctor,' and there should be every effort, wherever possible, to make this choice a reality. In no calling is there such a gap between perfunctory routine and the best endeavour, and the latter, in our opinion, would not be obtained under a whole-time State salaried service, which would tend, by its machinery, to discourage initiative, to diminish the sense of responsibility, and to encourage mediocrity."

